# Characterization of New Bioactive Enzyme Inhibitors from Endophytic *Bacillus amyloliquefaciens* RWL-1

**DOI:** 10.3390/molecules23010114

**Published:** 2018-01-05

**Authors:** Raheem Shahzad, Abdul Latif Khan, Liaqat Ali, Saqib Bilal, Muhammad Imran, Kyung-Sook Choi, Ahmed Al-Harrasi, In-Jung Lee

**Affiliations:** 1 School of Applied Biosciences, Kyungpook National University, Daegu 41566, Korea; raheemshehzad@ymail.com (R.S.); saqib043@yahoo.com (S.B.); m.imran02@yahoo.com (M.I.); 2 UoN Chair of Oman’s Medicinal Plants & Marine Natural Products, University of Nizwa, Nizwa 616, Oman; latifepm78@yahoo.co.uk (A.L.K.); malikhejric@gmail.com (L.A.); aharrasi@unizwa.edu.om (A.A.-H.); 3 Department of Chemistry, University of Sargodha, Sub-Campus Mianwali, Mianwali 42200, Pakistan; 4 School of Agricultural Civil & Bio-Industrial Machinery Engineering, Kyungpook National University, Daegu 41566, Korea; ks.choi@knu.ac.kr

**Keywords:** endophytes, biological potential, secondary metabolites, α-glucosidase, urease

## Abstract

Endophytic bacteria are known to produce a wide array of bioactive secondary metabolites with beneficial effects on human health. In the current study, a novel endophytic bacterial strain, *Bacillus amyloliquefaciens* RWL-1, was isolated from the seeds of Oryza sativa. Initially, the crude extract of RWL-1 was assessed for potential biological effects of enzyme inhibition and cytotoxicity and was found to exhibit a broad spectrum inhibition for α-glucosidase (37 ± 0.09%) and urease (49.4 ± 0.53%). The screening results were followed by bioassay-guided isolation of secondary metabolite(s) from RWL-1. Extensive chromatographic and spectrophotometry analyses revealed the presence of compound **1** (*S*)-2-hydroxy-*N*-((*S*)-1-((*S*)-8-hydroxy-1-oxoisochroman-3-yl)-3-methylbutyl)-2-((*S*)-5-oxo-2,5-dihydrofuran-2-yl)acetamide. Further bioassays of compound **1** showed significant inhibition of α-glucosidase (52.98 ± 0.8%) and urease (51.27 ± 1.0%), compared with positive control values of 79.14 ± 1.9% and 88.24 ± 2.2%, and negative controls (0.08 ± 0.1% and 0.05 ± 0.01%), respectively. The current study suggests that bacterial endophytes are a rich source of novel bioactive compounds with high therapeutic value.

## 1. Introduction

The term “endophyte” refers to microorganisms (e.g., bacteria, fungi) that live inside plant tissues without causing apparent diseases and have the ability to colonize the internal tissues of the plant [1,2]. Endophytes are pervasive in all plant parts and have been isolated from different plant parts [3]. They form mutualistic relationships with host plants, which are either obligatory or facultative associations, and do not damage the host plant; however, this mutualistic interaction also depends on many factors and can change over time [1,3,4,5]. In this extended symbiotic relationship, the plants provide shelter, protection, and access to essential nutrients; in return, the endophytes generate a beneficial correlation with the host through the modulation of endogenous phytohormones and nutrients, and improve the adaptability of plants to rapidly changing environments [6,7]. Although endophytes are present in all plant species, endophyte—host interaction has not been completely explored. This may include competitiveness in the host tissues mediated by the secretion of secondary metabolites and the detoxification of such inhibitors delivered by endophytes [8,9].

In addition to plant-microbe interactions, endophytes display many important biological activities [6,10]. Among endophytes, many endophytic bacteria are known to produce a diverse range of bioactive and health-promoting compounds, such as volatile organic compounds, antibiotics, immunosuppressant compounds, and anticancer, antiviral and antifungal drugs [11,12,13]. More than 60% of anticancer drugs in clinical use are natural products [14]. The first study of the endophytic bacteria *Bacillus amyloliquefaciens* afforded the discovery of an antitumor exopolysaccharide derived from the *Bacillus* genus [15]. In addition to antitumor compounds, the endophytes belonging to the *Bacillus* genus produce a variety of secondary metabolites; for example, *B. thuringiensis* produces the anti-infective compound anthracene [16] and *B. subtilis* produces the antimicrobial protein YbdN [17]. These results indicate the promising medicinal potential of the *Bacillus* genus. Owing to their involvement in phytostimulation, disease suppression, and other biological activities, *Bacillus* sp. are preferred because their long-term viability can facilitate the development of natural products suitable for commercial use [18].

*B. amyloliquefaciens* strains have attracted considerable interest because they are able to produce a wide range of active antimicrobial compounds, macrolactins, lipopeptides, hydrolytic enzymes, and certain volatile compounds. For example, *B. amyloliquefaciens* FZB42 has 8.5% of its genome dedicated to the synthesis of secondary metabolites [19], allowing the production of lipopeptides, surfactin, fengycin, bacillomycin D, polyketide (difficidin), dipeptide bacilysin, chitin, and colloidal chitin [20,21].

**Figure 1:**
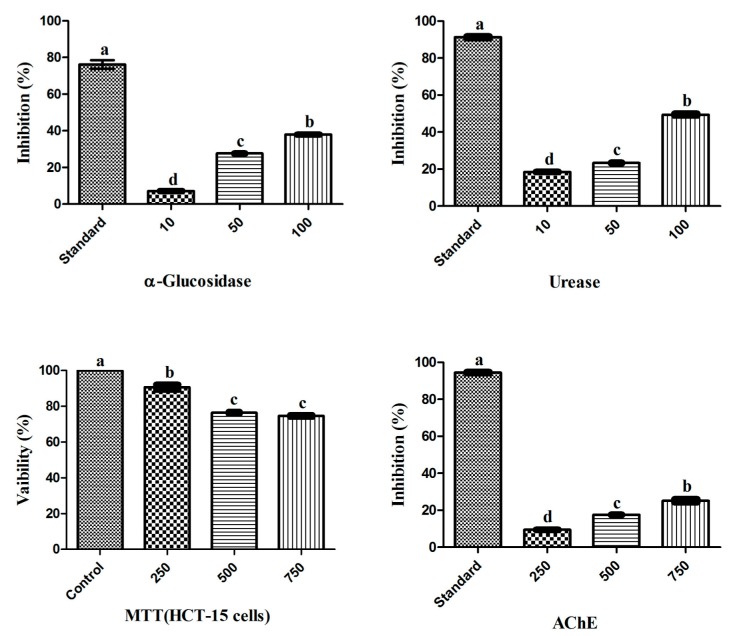
Biological potential of endophytic RWL-1 crude extract. The graph values are presented as the mean of three replicates with standard error. The acarbose, thiourea, DMSO and galantamine was used as reference standard for α-Glucosidase, Urease, MTT (HCT-15 cells) and AChE inhibition respectively. Bars with different letters are significantly different at *p* ≤ 0.05 based on Duncan multiple range test.

**Figure 2:**
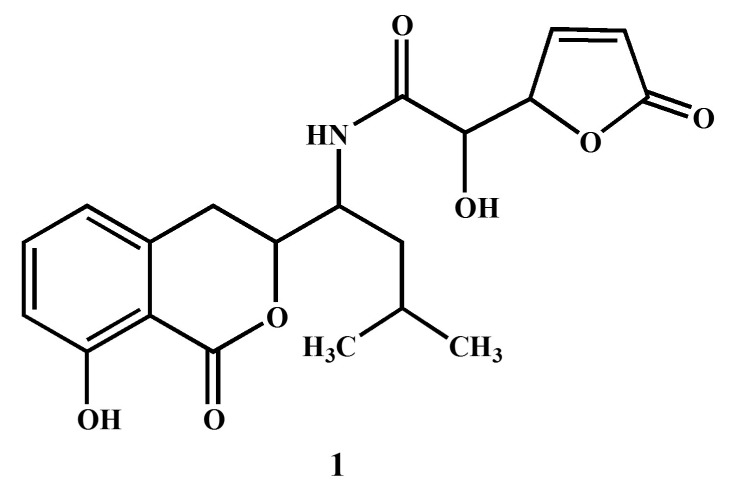
Nuclear magnetic resonance spectroscopic analysis of compound **1** ((*S*)-2-hydroxy-*N*-((*S*)-1-((*S*)-8-hydroxy-1-oxoisochroman-3-yl)-3-methylbutyl)-2-((*S*)-5-oxo-2,5-dihydrofuran-2-yl)acetamide).

*B. amyloliquefaciens* produce a variety of secondary metabolites, and previously, RWL-1 was isolated from rice seeds and was identified as *B. amyloliquefaciens*. RWL-1 was submitted to NCBI under the accession number (KR677384) and was reported to stimulate phytohormone and secondary metabolite production, promote plant growth, and ameliorate stress [1,22,23]. Therefore, given the medicinal potential of *B. amyloliquefaciens*, the current study aimed to explore the medicinal potential of RWL-1. The ethyl acetate extract of RWL-1 was subjected to biological assays for anticancer, anti-AChE, anti-α-glucosidase, and anti-urease activity (Figure 1). The positive screening results led to the assessment of RWL-1 by advanced chromatographic and NMR spectroscopic techniques to isolate the secondary metabolites responsible for α-glucosidase and urease inhibition. A secondary metabolite (Figure 2) was purified and the chemical structure was deduced from MS and NMR data analysis and comparison with the literature values [24]. The purified secondary metabolite was subjected to a urease inhibition bioassay to evaluate and confirm its medicinal potential.

## 2. Materials and Methods

### 2.1. Bacterial Growth Conditions

RWL-1 was grown in 8 L of LB medium (tryptone, 10 g; yeast extract, 5 g; sodium chloride, 10 g; pH 7.0 ± 0.2; autoclaved for 15 min at 121 °C). The inoculated RWL-1 was incubated on shaking incubator (120 rpm) for 7 days at 28 °C and centrifuged at 5000× *g* for 15 min to separate the cells from the culture broth.

### 2.2. Extraction for Secondary Metabolite Identification

The cell-free culture broth of RWL-1 was adjusted to pH 2.5 and was completely extracted three times with an equal volume of ethyl acetate (EtOAc). The ethyl acetate extract was then completely dried in a rotary evaporator to obtain the crude extract (1.6 g). The ethyl acetate crude extract was subjected to various biological assays for the assessment of its medicinal potential.

### 2.3. Secondary Metabolite Isolation

Based on the results of the bioassay, the ethyl acetate extract was analyzed by silica gel column chromatography using a solvent gradient (1% EtOAc/*n*-hexane to 85% EtOAc/*n*-hexane). TLC (pre-coated aluminum sheets, silica gel 60F-254, E. Merck, Darmstadt, Germany) experiments were performed to investigate and determine the different fractions of organic extract of bacterial culture broth. The TLC plates were observed under UV light at 254 and 365 nm (Vilber Lourmate, Marne-la-Vallee, France).

### 2.4. Reverse-Phase HPLC Analysis

To achieve further purification, the bioactive fractions were subjected to recycling preparative high-performance liquid chromatography (HPLC) using a Shimadzu device CBM-10 (Varian, Inc., Palo Alto, CA, USA) coupled with a UV-VIS detector (SPD-10A, SpectraLab Scientific Inc., Markham, ON, Canada). The sample was loaded onto a C_18_ column (Luna 5 µm, 100 Å, 250 mm × 4.60 mm) equipped with pumps A and B (LC-10AD). A solvent gradient of Solvent A—100% MeOH and Solvent B—Water with 5% acetic acid with Solvent program: 0–20 min (50% = A; 50% = B), 20–40 min (80% A, 20% B), 40–60 min (100% A, 0% B) was used at a flow rate of 1 mL/min. The remaining impurities were removed by loading the semi-pure secondary metabolite on preparative TLC plates (as described in “Secondary metabolite isolation”) and compound **1** (4.5 mg) was purified in DCM/*n*-hexane (95:5).

### 2.5. Structure Elucidation of Compound ***1***

The purified compound **1** was subjected to spectroscopic analyses (UV, IR, ^1^H-NMR, ^13^C-NMR, ESI, and MS/MS studies) for identification and characterization. The optical measurements were conducted by using a polarimeter (JASCO DIP360, Jasco Co., Tokyo, Japan). A Bruker ATR-Tensor 37 spectrophotometer (Bruker, Ettlingen, Germany) was used to record the IR spectra. To obtain the ESI mass spectra, a QSTAR mass spectrometer (Applied Biosystems, Foster, CA, USA) with capillary voltage of 5–5.5 kV was used. The NMR spectra (^1^H and ^13^C) were obtained by using a Bruker NMR spectrometer (Burker, Fallanden, Switzerland) operated at 600 MHz and 150 MHz, respectively.

### 2.6. In Vitro Biological Activities of RWL-1 Crude Extract

#### 2.6.1. Urease Inhibition

The inhibitory effects of the ethyl acetate crude extract and compound **1** on urease activity was measured in accordance with the method of [25]. Briefly, 100 mM urea (0.055 mL) in 8.2 pH phosphate buffer containing 0.01 M LiCl_2_, 0.1 mM EDTA, and 0.01 M K_2_HPO_4_·3H_2_O was reacted with 3 units/mL jack bean urease (0.025 mL) (Sigma, Munich, Germany) and various concentrations of crude extract (10–100 µg/mL) in a 96-well plate for 15 min at 37 °C. The urease inhibitory activity was evaluated through the measurement of ammonia production by the indophenol blue method. The absorbance was measured at 630 nm and thiourea was used as the standard inhibitor. The inhibition percentage was calculated from the following equation:

Inhibition (%) = 100 − (OD_test_/OD_control_) × 100 (1)

#### 2.6.2. α-Glucosidase Inhibition

The inhibitory effects of the ethyl acetate crude extract and compound **1** on α-glucosidase activity were measured in accordance with the method of [26]. Briefly, α-glucosidase was mixed with different concentrations of RWL-1 ethyl acetate crude extract and incubated for 10 min at 37 °C. Acarbose was used as a positive control and *p*-nitrophenyl α-d-glucopyranoside (PNP-G) was used as the substrate. The absorbance of *p*-nitrophenol released from *p*NPG was measured at 405 nm every 5 min. The inhibition percentage was calculated from the standard curve.

#### 2.6.3. Anticancer Assay (MTT)

The effect of the ethyl acetate crude extract and compound **1** on cell viability was evaluated by an MTT assay in accordance with the protocol described by [27]. Briefly, HCT-15 cells were purchased from American Type Culture Collection CCL-25 (USA) and were maintained at 37 °C in subconfluent conditions in a humidified atmosphere of 95% air and 5% CO_2_. RPM-1640 medium supplemented with 10% fetal bovine serum and 1% (*v*/*v*) streptomycin was used for subculturing. HCT-15 cells were subcultured at density of 10^5^ cells/well in 96-well plates with and without different concentrations (250, 500 and 750 µg/mL) of ethyl acetate crude extract of RWL-1 for 24, 48, and 72 h. The medium was removed and 20 µL MTT solution (5 mg/mL in PBS) was added to each well of the 96-well plate and incubated for 2 h at 37 °C. After incubation, the MTT medium was replaced with DMSO (200 µL). The plate was gently shaken for 1 min and the absorbance was measured at 540 nm. The following equation was used for the calculation of cell viability:

Viable cells (%) = (OD of treated sample/OD of untreated sample) × 100(2)

#### 2.6.4. AChE Inhibition

The AChE inhibition assay was conducted in accordance with the protocol described by [28] using a slightly modified version of Ellman’s colorimetric method. Briefly, 15 mM acetylthiocholine iodide in deionized water (25 µL) and 3 mM 5,5-dithiobis-2-nitrobenzoic acid (DTNB) (125 µL) in 50 mM Tris-HCl buffer, pH 8.0, containing 0.1 M NaCl and 0.02 M MgCl_2_∙6H_2_O, were added into a 96-well plate. Subsequently, 50 mM Tris-HCl buffer, pH 8.0, containing 0.1% bovine serum albumin (50 µL) and ethyl acetate crude extract (25 µL) were added at different concentration (50 µg/g to 600 µg/g). AChE (25 µL, 0.2 U/mL) was added and the absorbance was measured at 412 nm at 45 s intervals. Galantamine (0.5–5 µg/mL) was used as a standard inhibitor. The inhibition percentage was calculated from the following formula:

Inhibition (%) = 1 – (sample reaction rate/blank reaction rate) × 100(3)

### 2.7. Statistical Analysis

The data are presented as the mean ± S.D. of three replicate experiments. Graphical representations was computed by using GraphPad Prism software 6.01 package (GraphPad Software, Inc., La Jolla, CA, USA) and statistical analyses were conducted with Duncan’s multiple range test in SAS version 9.2 (Cary, NC, USA).

## 3. Results and Discussion

### 3.1. Biological Potential of RWL-1 Crude Extract

Throughout history, natural products have consistently been the best source for prominent compounds with a potent role in the fields of agriculture, medicine, and pharmacy [29,30]. Among natural products, microorganisms are considered to contain an abundance of unlimited bioactive metabolites with a high therapeutic value [31]. Recently, scientists have focused on endophytes because of the unique ecological niche these organisms live in [32] and several bioactive compounds with compelling therapeutic applications, such as anticancer, anti-obesity, anti-AChE, and anti-α-glucosidase enzymes have been isolated from endophytes [15,16,26,33]. However, the isolation, purification, and identification of potent bioactive compounds are particularly laborious and difficult [34]. Therefore, in the current study, the crude extract of endophytic *B. amyloliquefaciens* RWL-1 was screened for its biological potential.

The biological potential of the RWL-1 crude extract was examined through its inhibitory activity on various enzymes and cytotoxicity (Figure 1). The inhibition of α-glucosidase, urease, AChE, and the cytotoxicity of cancerous HCT-15 cells was examined in response to treatment with various concentrations of the RWL-1 crude extract; significant inhibition of α-glucosidase and urease was observed, but no significant reduction of AChE activity or HCT-15 cell viability was found (Figure 1).

The crude extract showed inhibition of α-glucosidase and urease as the concentration of RWL-1 crude extract increased (10–100 µg/mL). A higher dose (100 µg/mL) significantly inhibited α-glucosidase (37 ± 0.09%) and urease (49.4 ± 0.53%), with the positive control leading to 74.85 ± 0.06% and 90.86 ± 0.08% inhibition, respectively. The cytotoxicity and AChE inhibition of ethyl acetate crude extract of RWL-1 were determined to occur in a dose-dependent manner at relatively high doses (250–750 µg/mL). The inhibition of cell growth was examined after exposure to different concentrations of the ethyl acetate crude extract of RWL-1. The results revealed that the RWL-1 crude extract showed a small cytotoxic effect (25 ± 0.16%) at a higher concentration (750 µg/mL) compared with the control (100%). A similar trend was also observed for AChE inhibition. No significant decreases were observed in the AChE activity in response to different concentrations (250–750 µg/mL) of the RWL-1 crude extract, although the positive control compound significantly inhibited AChE (94.45 ± 0.31%).

### 3.2. Structural Elucidation of Compound ***1***

The structure elucidation of compound **1** was conducted by the analysis of NMR and MS spectral data in comparison with the data reported in previous studies [24,35,36]. The 1H-NMR spectrum displayed signals for five protons in the aromatic region (δ 8.76–7.59) owing to tri-substituted benzene and the unsaturated γ-lactone moieties in the molecule. A broad singlet in the downfield region at δ 9.15 was assigned to the amide proton NH in the molecule. In addition to these aromatic signals, three oxy-methine protons (δ 4.24–3.84) and two methyl groups (6H, d, *J* = 7.4 Hz) were also observed in the ^1^H-NMR spectrum. In the ^13^C-NMR spectrum, the unsaturated γ-lactone was indicated by the presence of a carbonyl carbon at δ 176.2, in addition to two characteristic sp^2^ methine signals at δ 153.7 and 132.4. The aromatic methine carbons of the substituted benzene ring appeared at δ 139.3, 129.9, and 125.3. The spectral data of compound **1** (Name: (*S*)-2-hydroxy-*N*-((*S*)-1-((*S*)-8-hydroxy-1-oxoisochroman-3-yl)-3-methylbutyl)-2-((*S*)-5-oxo-2,5-dihydrofuran-2-yl)acetamide) were further compared with those reported in literature [24,35,36]. The overall physical and spectral data of compound **1** were found to be identical to the reported antibiotic Al 77F, which was previously isolated from bacterial strains of *Bacillus pumilus* [36] and the fungal strains of *Alternaria tenuis* [24].

### 3.3. Biological Evaluation of Compound ***1***

**Figure 3:**
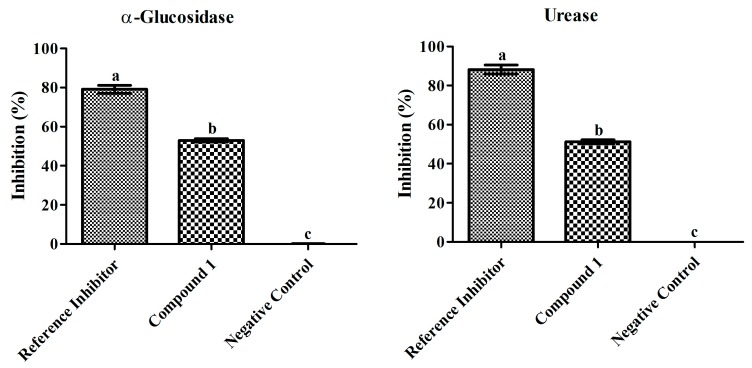
Inhibitory effects of compound **1** (100 µg mL^−1^) and reference inhibitors (80 µg mL^−1^) on α-glucosidase and urease. The graph values are standard error of means of three replications. The acarbose and thiourea was used as reference inhibitor for α-glucosidase and urease. Bars with different letters are significantly different at *p* ≤ 0.05 based on Duncan multiple range test.

Based on the biological potential of the crude extract, the enzyme inhibition potential of compound **1** was evaluated. The inhibition patterns of α-glucosidase and urease in response to compound **1** are shown in Figure 3.

The α-glucosidase inhibition efficiency of compound **1** was examined at different doses and the inhibition percentage significantly increased as the concentration increased (10–100 µg/mL) showing 94.37 µg/mL IC_50_ value. The highest concentration of compound **1** (100 µg/mL) resulted in the strongest α-glucosidase inhibition (52.98 ± 0.8%). However, the standard drug used as the positive control (10–100 µg/mL) resulted in highest inhibition (79.14 ± 1.9%) at 80 µg/mL displaying an IC_50_ value of 62.03 µg/mL, while the negative control resulted in 0.08 ± 0.1% inhibition.

The urease inhibition was also evaluated at different doses and the inhibition percentage increased as the concentration of compound **1** increased (10–100 µg/mL) exerting an IC_50_ value of 97.52 µg/mL. The highest dose of compound **1** (100 µg/mL) marked significant inhibition (51.27 ± 1.0%) and the standard drug used as the positive control resulted in 88.24 ± 2.2% at 80 µg/mL with 55.13 µg/mL IC_50_ value, while the negative control resulted in 0.05 ± 0.01% inhibition.

In recent years, interest has intensified in the isolation and identification of bioactive α-glucosidase and urease inhibitors that can both be utilized as tools to comprehend biochemical processes and function as prospective therapeutic agents [26,33]. Although plants are a vital source of bioactive metabolites, microbes are also considered an important source of bioactive metabolites with high therapeutic value [37,38]. Although the biological potential of *B. amyloliquefaciens* is widely reported [15], this study is the first to describe the enzyme inhibitory activity of *B. amyloliquefaciens* and the bioactive compounds isolated from this species. Moreover, the biological potential of isolated compound **1** from *B. amyloliquefaciens* RWL-1 has been reported to have anti-bacterial and antiulcer properties [39,40]. The results of the current study are in agreement with the findings of other researchers who have reported that endophytes provide plentiful resources from which α-glucosidase and urease inhibitors can be found [26,33].

## 4. Conclusions

**Figure 4:**
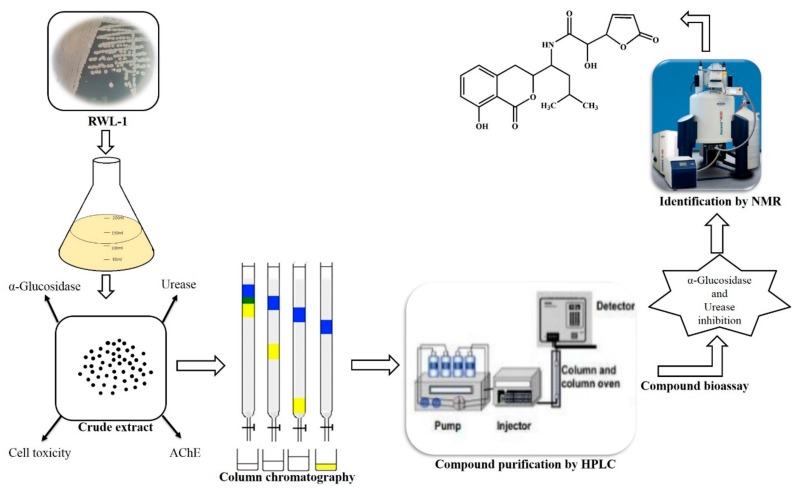
Schematic representation of biological potential of endophytic *B. amyloliquefaciens* RWL-1 and bioassay guided isolation of bioactive metabolite (Compound **1**) for α-glucosidase and urease inhibition.

The current study evaluated the biological potential of endophytic *B. amyloliquefaciens* RWL-1 and conducted bioassay-guided isolation of bioactive metabolites responsible for enzymes inhibition (Figure 4). The screening of isolated compound **1** (*S*)-2-hydroxy-*N*-((*S*)-1-((*S*)-8-hydroxy-1-oxoisochroman-3-yl)-3-methylbutyl)-2-((*S*)-5-oxo-2,5-dihydrofuran-2-yl)acetamide supported the observed effects of *B. amyloliquefaciens* RWL-1 in α-glucosidase and urease inhibition. *B. amyloliquefaciens* is considered an ecologically and economically valuable source for the production of bioactive metabolites. Thus, the current study suggests the importance of endophytic microbes in the search for natural bioactive metabolites with high therapeutic potential.
